# Pseudo-Bladder and Double-Bladder Signs Suggestive of Pediatric Ovarian Torsion Associated With Pelvic Cystic Lesions: A Case Series

**DOI:** 10.7759/cureus.97888

**Published:** 2025-11-26

**Authors:** Takao Komaru, Keiichi Tomita, Mikiko Miyasaka, Akihiro Yoneda, Akira Ishiguro

**Affiliations:** 1 Department of Pediatrics, National Center for Child Health and Development, Tokyo, JPN; 2 Department of Emergency and Transport Medicine, National Center for Child Health and Development, Tokyo, JPN; 3 Department of Radiology, National Center for Child Health and Development, Tokyo, JPN; 4 Department of Surgery, National Center for Child Health and Development, Tokyo, JPN; 5 Department of Pediatrics, National Hospital Organization Disaster Medical Center, Tokyo, JPN

**Keywords:** adnexal torsion, double-bladder sign, ovarian cyst, ovarian torsion, pediatric, pediatric emergency medicine, pocus, point-of-care ultrasound, pseudo-bladder sign, ultrasound

## Abstract

Ovarian torsion is a rare but serious cause of acute abdominal pain in female children, and delayed diagnosis can result in infertility. In this case series of five pediatric patients, we report a case in which point-of-care ultrasound demonstrated a unique finding that we refer to as the “pseudo-bladder sign.” In this case, a collapsed bladder led to the misidentification of an ovarian cyst as the bladder, contributing to a delayed diagnosis of ovarian torsion. To provide a clearer understanding of this phenomenon, we additionally present four cases in which radiology-performed ultrasound demonstrated the “double-bladder sign,” where a cyst simulated a second bladder. Although these signs indicate the presence of a pelvic cyst rather than torsion itself, they may serve as a diagnostic clue for ovarian torsion when accompanied by acute abdominal pain. Recognition of these findings, combined with accurate knowledge of female pelvic anatomy and systematic scanning in transverse and longitudinal planes, may facilitate earlier diagnosis, help prevent diagnostic delays, and potentially reduce the risk of ovarian loss.

## Introduction

Ovarian torsion is a rare but serious cause of acute abdominal pain in children, and delayed diagnosis may result in infertility [1]. Clinically, the presentation of ovarian torsion is often nonspecific, with symptoms such as abdominal pain, nausea, and vomiting, which can contribute to diagnostic difficulty [2,3].

Ultrasound is the first-line imaging modality because it is noninvasive and free of radiation exposure [2,4,5]. A characteristic sonographic finding, the “double-bladder sign,” in which an ovarian cyst appears adjacent to the bladder and simulates a second bladder, has been reported in adults as a suggestive finding of ovarian torsion associated with ovarian cysts [6,7]. The double-bladder sign essentially represents the presence of a pelvic cyst rather than ovarian torsion. However, in female patients presenting with acute abdominal pain, this finding should still prompt consideration of ovarian torsion. To date, no such reports have been described in children.

In this report, we describe a pediatric case in which a collapsed bladder on point-of-care ultrasound (POCUS) led to the misidentification of an ovarian cyst as the bladder, contributing to a delayed diagnosis of ovarian torsion. We refer to this finding as the “pseudo-bladder sign.” To further enhance understanding of this phenomenon, we also describe four additional pediatric cases in which radiology-performed ultrasound (RADUS) demonstrated the double-bladder sign.

This study was previously presented at the 52nd Annual Meeting of the Japanese Association for Acute Medicine, held on October 15, 2024.

## Case presentation

Case 1

**Figure 1:**
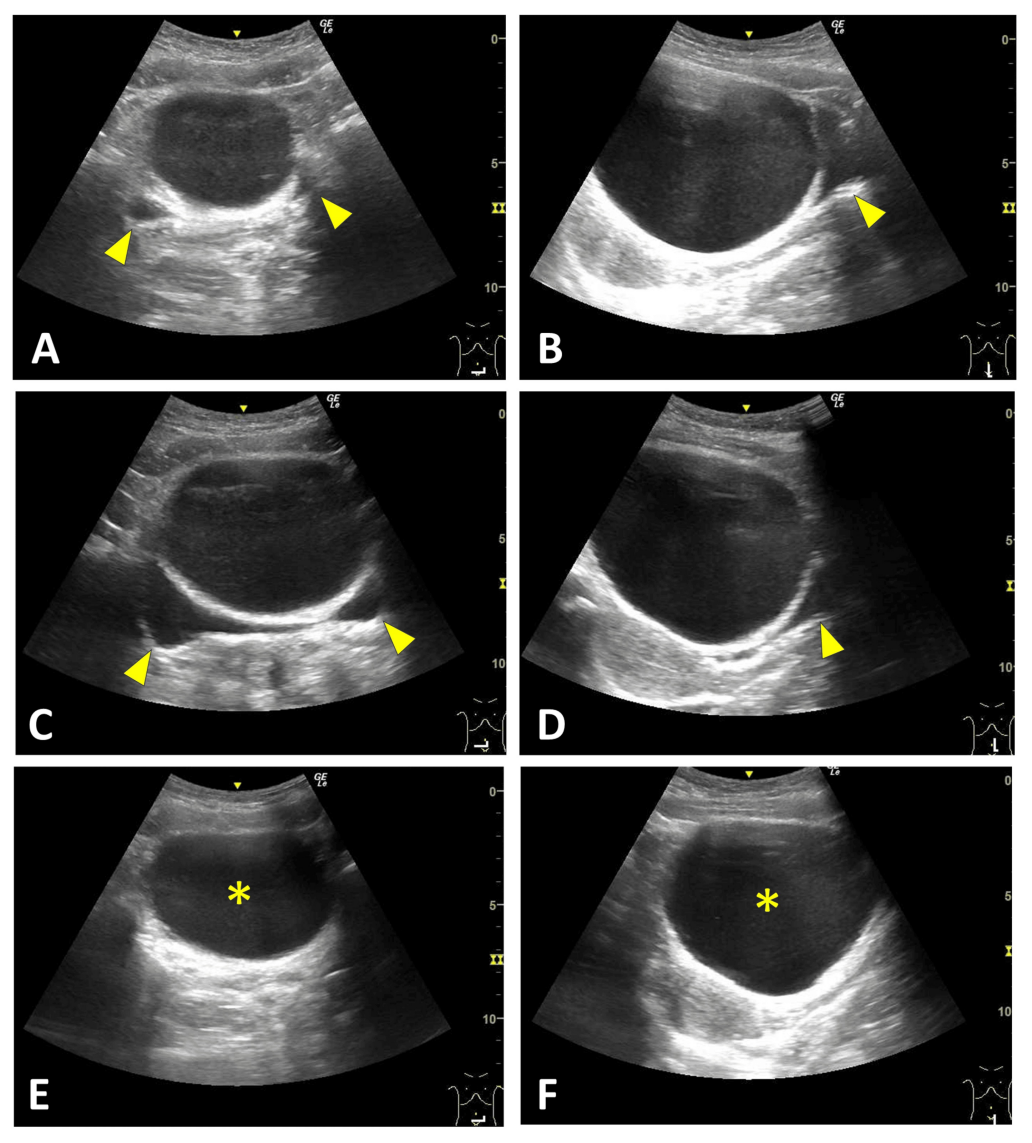
Cystic Pelvic Mass Mimicking the Urinary Bladder on POCUS Initial POCUS demonstrated an anechoic area in the transverse (A) and longitudinal (B) views, which was initially interpreted as ascites (arrowheads). Repeat prevoid scans showed an enlarged anechoic area (C, D; arrowheads). Subsequent postvoid scans confirmed that the region initially presumed to be the bladder was actually a cystic mass (*) (E, F). POCUS: point-of-care ultrasound

**Figure 2:**
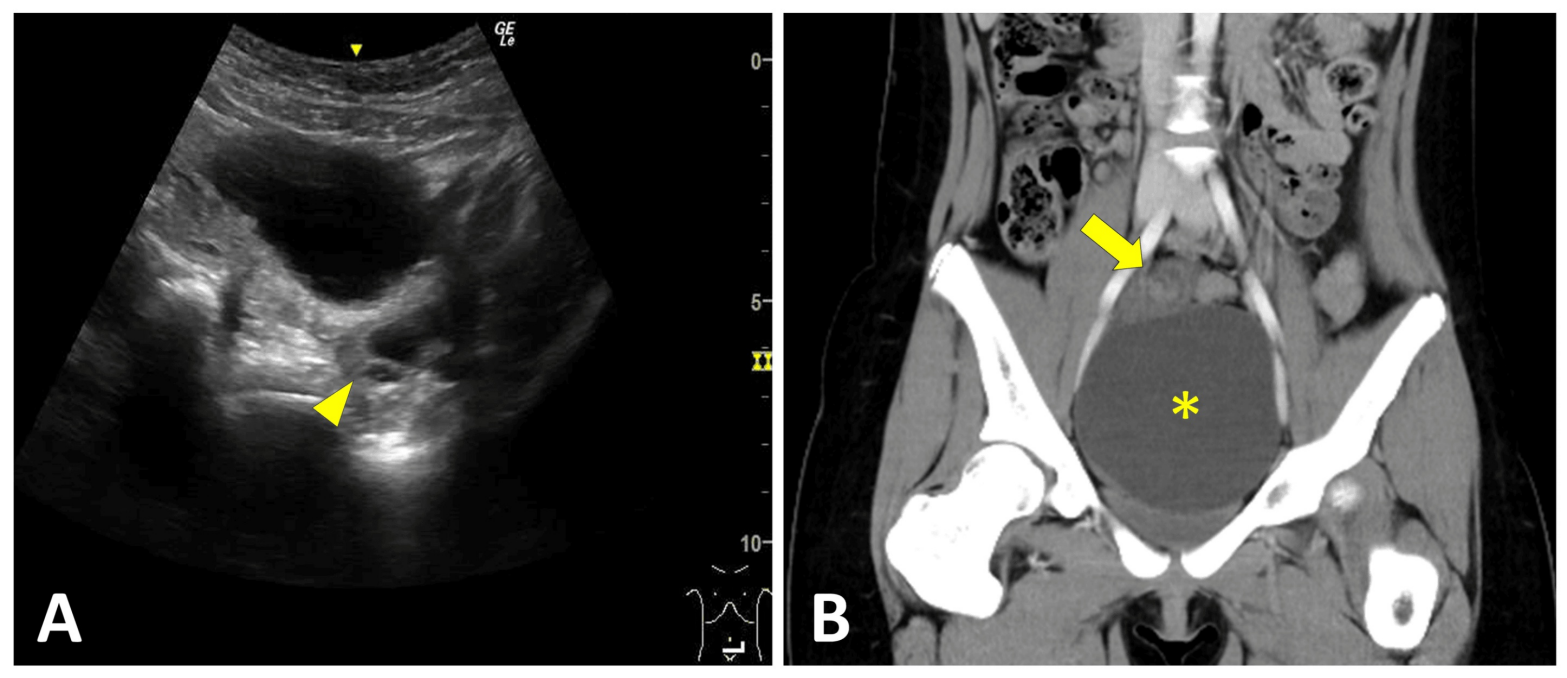
Normal Left Ovary on POCUS and Enlarged Right Adnexa With Whirlpool Sign on CT Transverse POCUS (A) demonstrated a normal left ovary with a follicular pattern (arrowhead). Coronal CT (B) demonstrated an enlarged right adnexa with a whirlpool sign (arrow) superior to a cystic mass (*). POCUS: point-of-care ultrasound

A 12-year-old girl presented with a 12-hour history of lower abdominal pain. Vital signs were as follows: body temperature, 36.4°C; heart rate, 100 beats/min; and respiratory rate, 20 breaths/min. Physical examination revealed mid-lower abdominal tenderness without rebound or a palpable mass. Initial POCUS, performed by a physician in training under the direct supervision of a pediatric emergency physician authorized to perform POCUS independently, using a 2 to 5 MHz convex probe, demonstrated an anechoic pelvic area interpreted as physiologic ascites (Figures 1A, 1B). Although no significant stool burden was seen on POCUS, a rectal enema was administered in light of her history of constipation. This resulted in a bowel movement and relief of her symptoms, after which she was discharged. However, she returned five hours later with recurrent pain and vomiting. Repeat POCUS, performed by a physician in training under the direct supervision of a pediatric emergency physician, demonstrated an enlarged anechoic area compared with the archived static images from the initial scan (Figures 1C, 1D). Prevoid and postvoid scans revealed that the presumed bladder was actually a 90-mm cystic lesion without septations or solid components (Figures 1E, 1F). A subsequent POCUS examination focused on the pelvic reproductive organs, performed under the supervision of a pediatric radiologist, demonstrated a normal left ovary, whereas the right ovary was not visualized (Figure 2A). As these bedside evaluations did not yield a definitive diagnosis, contrast-enhanced computed tomography (CT) was obtained, which demonstrated an enlarged right adnexa with a whirlpool sign (Figure 2B). Emergency surgery confirmed a 360° right ovarian torsion, and detorsion was performed with ovarian preservation and cystectomy. The patient recovered uneventfully and was discharged on hospital day 4. Pathological examination revealed a serous cystadenoma.

Case 2

**Figure 3:**
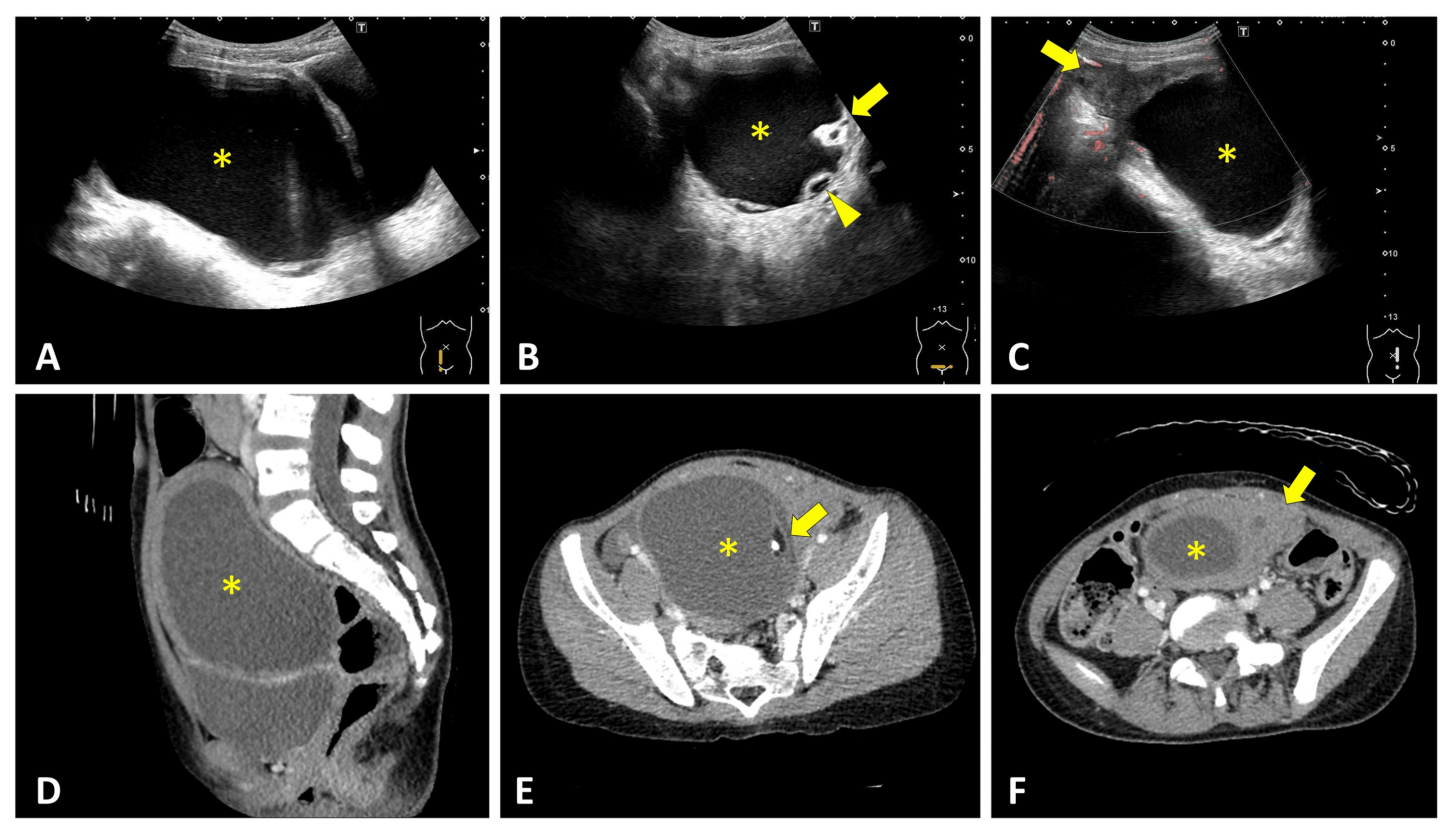
Double-Bladder Sign on RADUS With Corresponding CT Findings Longitudinal RADUS (A) demonstrated a cystic mass (*) producing the double-bladder sign. The transverse view (B) depicted a daughter cyst sign (arrowhead) and calcification (arrow). Color Doppler ultrasound showed partial flow within the enlarged left ovarian lesion (C, arrow). Sagittal CT (D) demonstrated a large cystic mass (*) consistent with the double-bladder sign observed on ultrasound. Axial CT revealed calcification and fat (E, arrow) within the cystic mass (*) and an enlarged left ovary with follicles (F, arrow). RADUS: radiology-performed ultrasound

An eight-year-old girl presented with a three-day history of lower abdominal pain and vomiting. Vital signs were as follows: body temperature, 38.5°C; heart rate, 120 beats/min; and respiratory rate, 20 breaths/min. Physical examination revealed diffuse abdominal tenderness, more pronounced on the left, without rebound tenderness or a palpable mass. RADUS, performed by a pediatric radiologist using a 4-6 MHz convex probe, demonstrated a 110-mm cystic lesion superior to the bladder, producing the double-bladder sign (Figure 3A). The lesion contained calcification and small peripheral cysts, depicting a daughter cyst sign (Figure 3B). The right ovary appeared normal. Color Doppler ultrasound showed partial flow within the enlarged left ovarian lesion, with leftward uterine deviation (Figure 3C). Contrast-enhanced CT demonstrated a large cystic lesion superior to the bladder containing calcification and fat (Figures 3D, 3E). Superior and to the left of this lesion, an enlarged left ovary with follicles was identified (Figure 3F). Emergency surgery confirmed a 630° left ovarian torsion, and detorsion was performed with ovarian preservation and cystectomy. Pathological examination confirmed a mature teratoma.

Case 3

**Figure 4:**
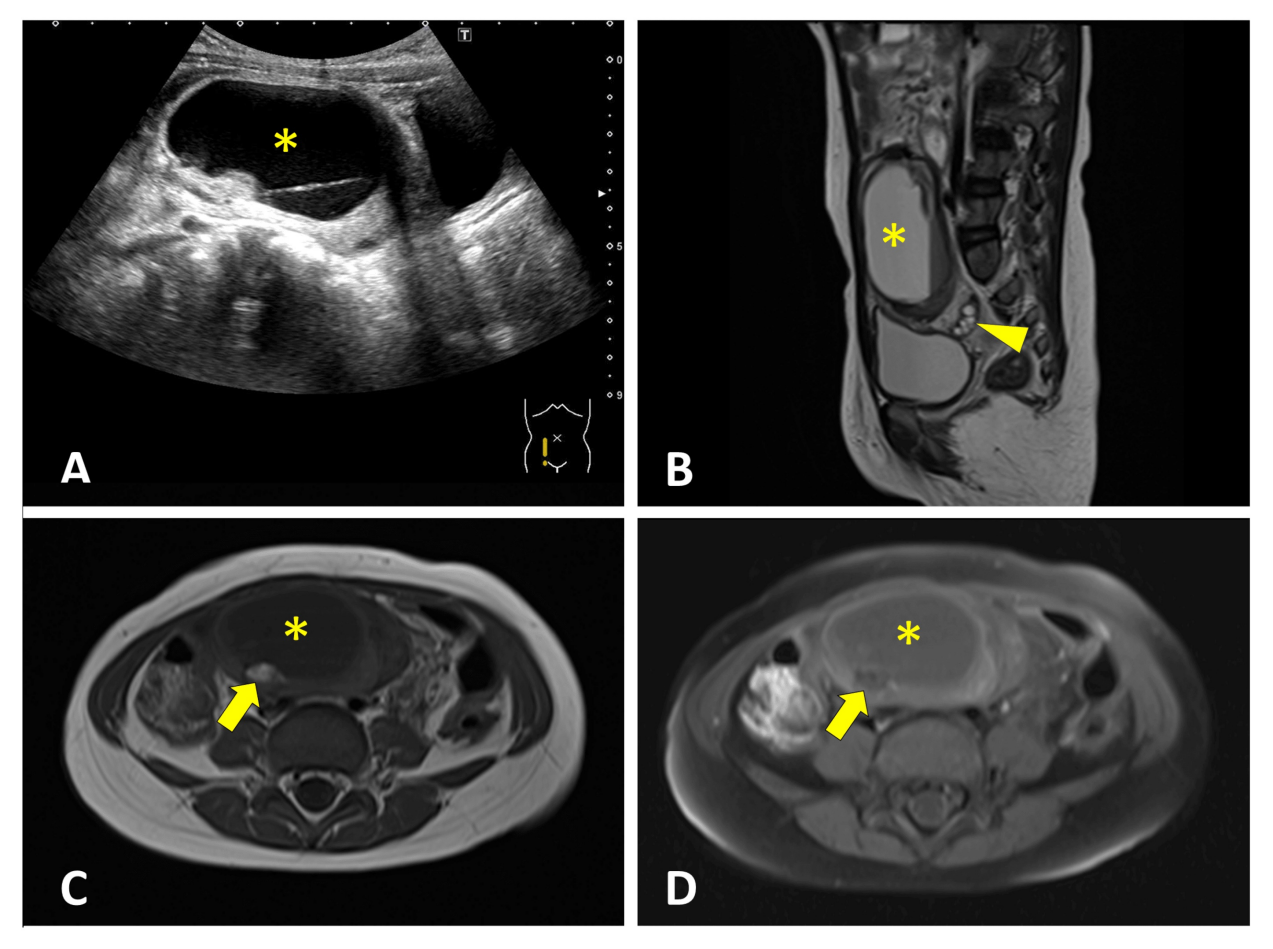
Double-Bladder Sign on RADUS With Corresponding MRI Findings Longitudinal RADUS (A) demonstrated a cystic mass (*) producing the double-bladder sign. Sagittal T2-weighted MRI (B) demonstrated a corresponding large cystic lesion (*), with the normal right ovary located posterior to the lesion (arrowhead). Axial T1-weighted and T1 fat-suppressed images (C, D) suggested the presence of intralesional fat (arrow). RADUS: radiology-performed ultrasound

A three-year-old girl presented with a four-day history of abdominal pain and vomiting. She was referred to our hospital after an ultrasound examination at a previous hospital suggested an ovarian tumor. Vital signs were as follows: body temperature, 38.5°C; heart rate, 140 beats/min; respiratory rate, 24 breaths/min; and blood pressure, 80/57 mm Hg. Physical examination revealed abdominal distension without definite tenderness, rebound tenderness, or a palpable mass. RADUS, performed by a pediatric radiologist using a 4-6 MHz convex probe, demonstrated a 58-mm cystic lesion superior to the bladder, producing the double-bladder sign (Figure 4A). The lesion contained solid components and a fluid-fluid level. The right ovary appeared normal, whereas the left ovary was not visualized. Magnetic resonance imaging (MRI) demonstrated a large cystic lesion with the normal right ovary located posterior to the lesion (Figure 4B). MRI signal characteristics were indicative of intralesional fat (Figures 4C, 4D). These findings were highly suggestive of left ovarian torsion due to a teratoma. Emergency surgery confirmed a 360° left ovarian torsion, and detorsion was performed with ovarian preservation and cystectomy. Pathological examination confirmed a mature cystic teratoma.

Case 4

**Figure 5:**
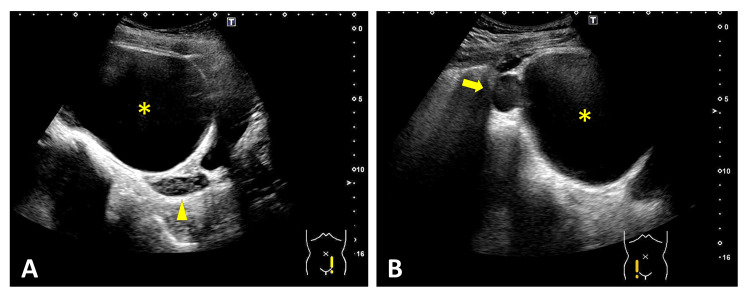
Double-Bladder Sign on RADUS Longitudinal RADUS (A) demonstrated a cystic mass (*) producing the double-bladder sign, with the normal left ovary (arrowhead) identified posterior to the cystic lesion. A mass with a daughter cyst sign was seen superior to the lesion on the right side (B, arrow). RADUS: radiology-performed ultrasound

A 14-year-old girl presented a few hours after the onset of abdominal pain and vomiting. Vital signs were as follows: body temperature, 36.2°C; heart rate, 78 beats/min; and respiratory rate, 20 breaths/min. Physical examination revealed lower abdominal distension, and the patient appeared to be in severe pain with a grimacing expression. No palpable mass was detected. RADUS, performed by a pediatric radiologist using a 4-6 MHz convex probe, demonstrated a 111-mm cystic lesion superior to the bladder, producing the double-bladder sign (Figure 5A). The normal left ovary was identified posterior to the cystic lesion (Figure 5A). A mass with a daughter cyst sign was seen superior to the lesion on the right side (Figure 5B), and, in conjunction with the clinical findings, these imaging features strongly suggested right ovarian torsion. Emergency surgery confirmed a 360° right ovarian torsion, and detorsion was performed with ovarian preservation and cystectomy. Pathological examination revealed an immature teratoma.

Case 5

**Figure 6:**
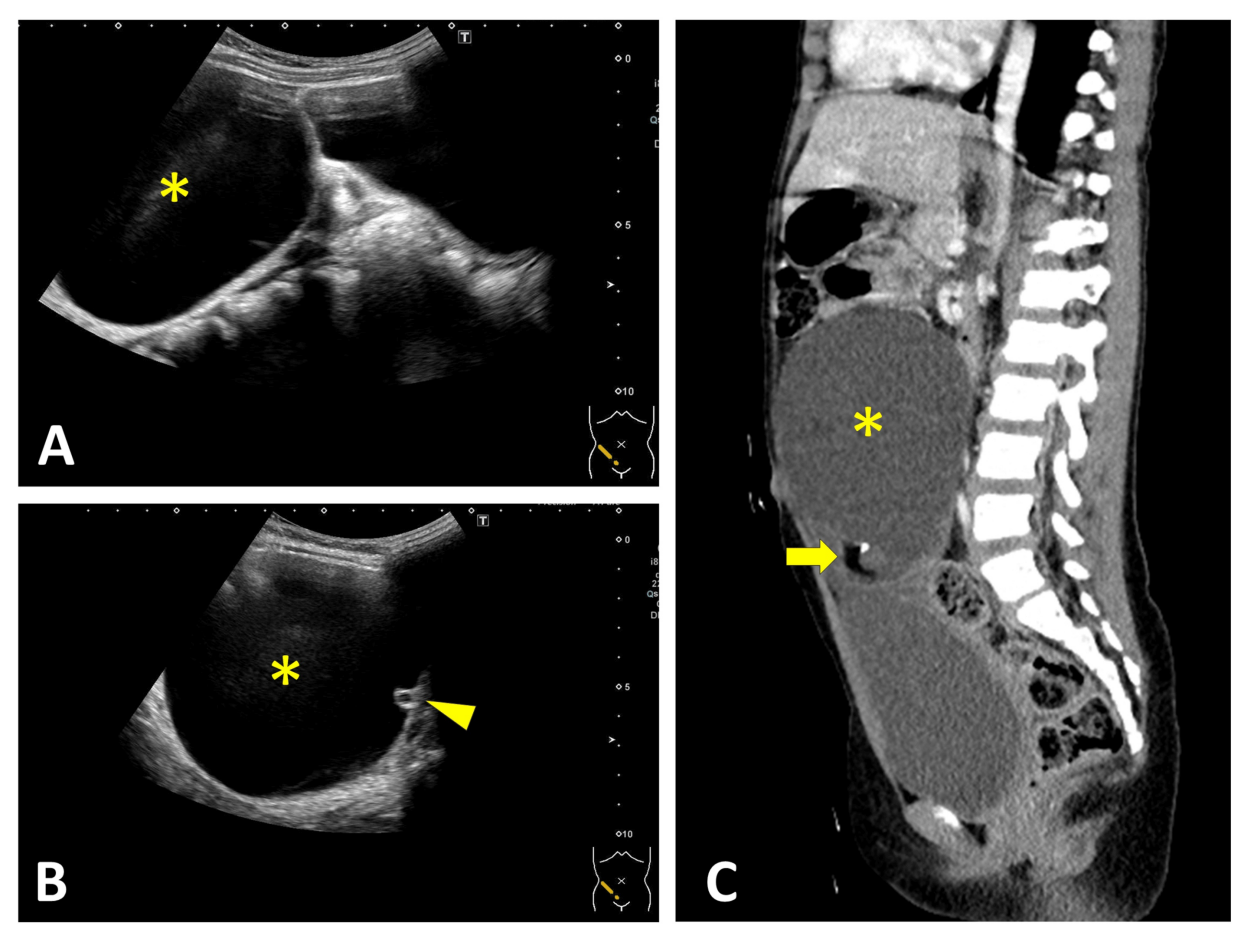
Double-Bladder Sign on RADUS With Corresponding CT Findings Longitudinal RADUS (A) demonstrated a cystic mass (*) producing the double-bladder sign and depicting a daughter cyst sign (B, arrowhead). CT demonstrated a cystic mass (*) containing calcification and fat (C, arrow). RADUS: radiology-performed ultrasound

A five-year-old girl presented with a six-day history of abdominal pain. Vital signs were as follows: body temperature, 36.7°C; heart rate, 84 beats/min; and respiratory rate, 20 breaths/min. Physical examination revealed a firm, elastic mass in the right lower abdomen, with localized tenderness over the same area. RADUS, performed by a pediatric radiologist using a 4-6 MHz convex probe, demonstrated a 117-mm cystic mass superior to the bladder on the right side, producing the double-bladder sign (Figure 6A) and depicting a daughter cyst sign (Figure 6B). CT demonstrated a cystic mass containing calcification and fat (Figure 6C), findings consistent with right ovarian torsion due to a teratoma. Emergency surgery confirmed a 360° right ovarian torsion, and detorsion was performed with ovarian preservation and cystectomy. Pathological examination confirmed a mature teratoma.

## Discussion

Ovarian torsion requires rapid diagnosis and emergency surgery to preserve ovarian function [1]. Although rare in patients under 20 years of age, with approximately 4.9 cases per 100,000 persons, delayed diagnosis increases the risk of ovarian necrosis and subsequent infertility [1]. As the clinical presentation is often nonspecific, with symptoms such as abdominal pain, nausea, and vomiting, diagnostic delays occur in up to 30% of cases [2,3]. Given these diagnostic challenges, imaging plays a pivotal role in achieving early detection.

Ultrasound is the first-line imaging modality for suspected ovarian torsion because it is noninvasive, free of radiation exposure, and readily available in pediatric emergency settings [2,4,5]. Reported sensitivity and specificity are 79% and 76%, respectively, with modest improvement when combined with color Doppler [5]. Findings such as stromal edema, reduced blood flow, and the whirlpool sign are highly specific for ovarian torsion, whereas adnexal masses and pelvic free fluid are less accurate [8]. In addition, a study conducted in a tertiary obstetrics and gynecology ultrasound unit, where five senior ultrasound specialists performed all examinations, demonstrated that diagnostic accuracy ranges widely (60% to 100%) and is highly operator-dependent [9].

For second-line imaging, CT or preferably MRI may be considered [4]. With contrast enhancement, both CT and MRI allow assessment of key findings such as reduced or absent enhancement of the ovarian stroma, associated hemorrhage or necrosis, and visualization of a swirling appearance of a twisted pedicle (whirlpool sign) [4]. Although CT can be performed rapidly in emergency settings [4], its sensitivity for diagnosing ovarian torsion is relatively low (42.2%) [10]. MRI offers the highest diagnostic accuracy among available imaging modalities, with reported sensitivity and specificity of 81% and 91%, respectively [5]. However, its use in emergent settings may be limited by equipment availability and the potential need for sedation in pediatric patients.

In the context of POCUS, reports of pediatric ovarian torsion remain limited to case reports and small case series [2,11]. The “double-bladder sign,” observed on POCUS as an ovarian cyst adjacent to the bladder and mimicking a second bladder, has been described in adults as an imaging clue suggestive of ovarian torsion associated with ovarian cysts [6,7]. However, this finding has not been well documented in children. Furthermore, to our knowledge, pediatric cases in which a collapsed bladder led to the misinterpretation of an ovarian cyst as the bladder have not been previously reported.

In our series, two characteristic sonographic signs were identified. Four patients demonstrated the double-bladder sign on RADUS (Cases 2 to 5, Figures 3-6). In contrast, one case evaluated with POCUS showed the misidentification of an ovarian cyst as the bladder due to bladder collapse during the initial scan (Case 1, Figure 1). We herein refer to this finding as the “pseudo-bladder sign."

**Figure 7:**
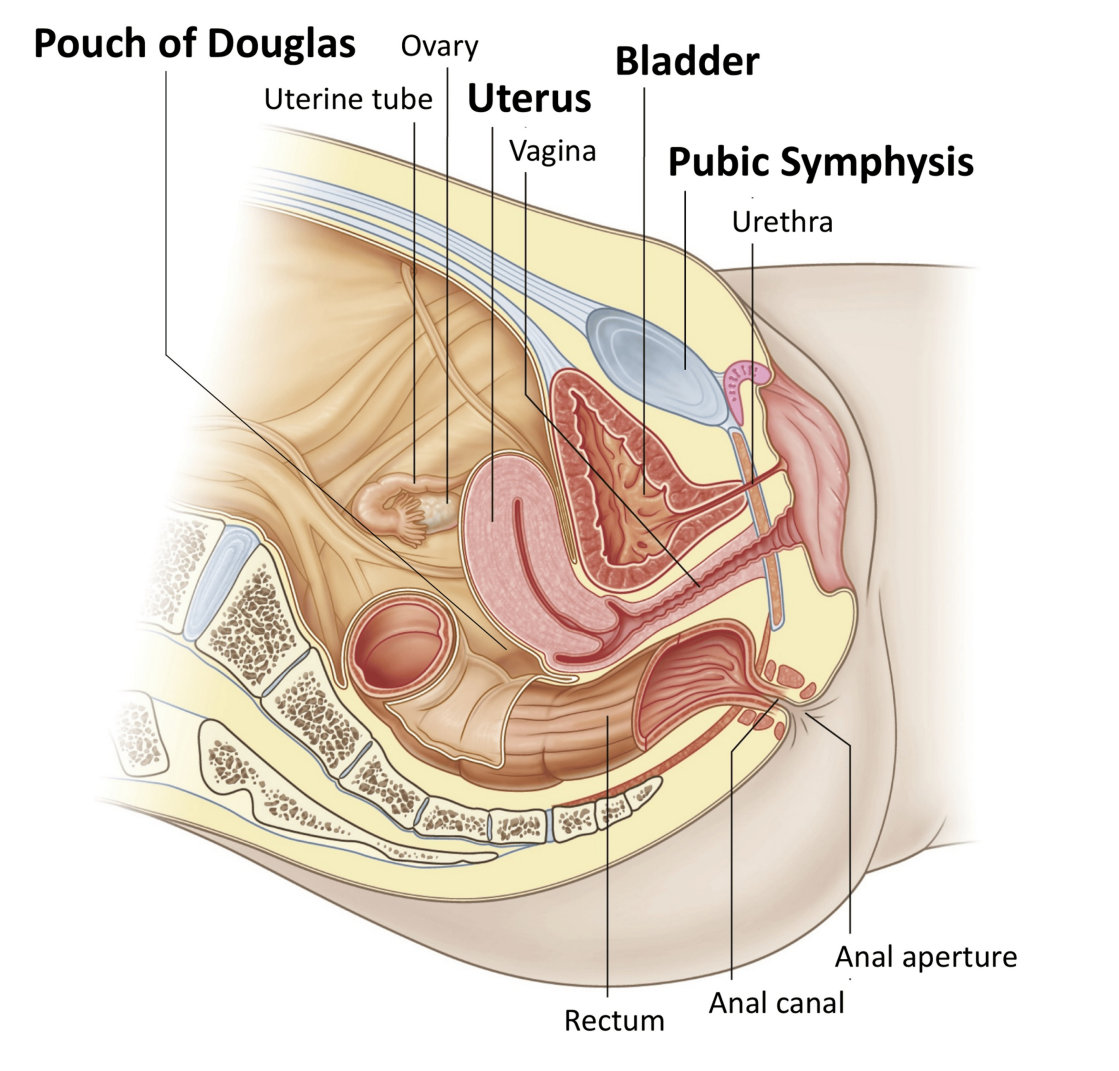
Schematic Overview of the Normal Female Pelvis From this schematic, it can be seen that the fluid closest to the pubic symphysis represents the bladder rather than peritoneal fluid (including the pouch of Douglas). Adapted from *Gray’s Anatomy for Students*, Drake RL, Vogl AW, Mitchell AWM, Fig. 5.2A, p. 411, Copyright © 2024, with permission from Elsevier.

**Figure 8:**
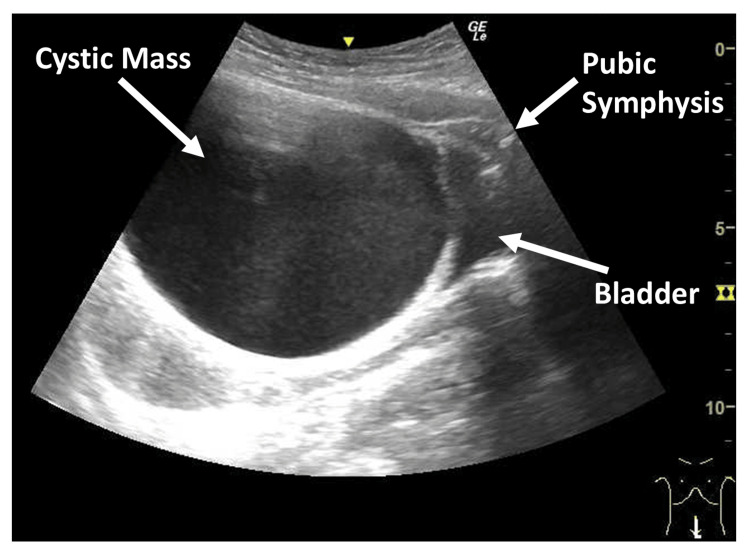
Relationships on Initial Longitudinal POCUS in Case 1: Distinguishing the Bladder From a Cystic Mass On the initial longitudinal POCUS in Case 1, the cystic mass appeared to mimic the bladder. Recognizing that the bladder is the fluid-filled structure closest to the pubic symphysis, the fluid adjacent to the symphysis can be accurately identified as the bladder.

In the case demonstrating the pseudo-bladder sign (Case 1), the cystic structure was misinterpreted as the bladder on the initial POCUS examination. A retrospective review of the stored longitudinal image yielded instructive findings that highlighted the importance of accurately understanding normal pelvic anatomy in females (Figure 1B). Anatomically, on longitudinal imaging, the bladder lies immediately posterior to the pubic symphysis, the uterus is located posterior to the bladder, and the rectouterine pouch (pouch of Douglas) lies further posteriorly (Figure 7). Had these anatomic landmarks been more clearly appreciated during the initial POCUS examination, the anechoic structure closest to the pubic symphysis might have been more readily recognized as the bladder rather than as peritoneal fluid (Figure 8). In addition, systematic scanning in both transverse and longitudinal planes is essential for adequately identifying these anatomic landmarks.

In clinical practice, the significance of a double- or pseudo-bladder sign depends on the patient’s clinical presentation. In female pediatric patients presenting with acute abdominal pain, the presence of either sign should prompt urgent evaluation for possible ovarian torsion [2,6,7]. When POCUS does not provide a definitive diagnosis, further specialist evaluation, including RADUS, is warranted. When necessary, second-line imaging with CT or preferably MRI should be considered, provided that it does not delay potential surgical management [4]. In contrast, in patients without acute symptoms, a double- or pseudo-bladder sign is more likely to reflect a pelvic cyst than ovarian torsion.

## Conclusions

This case series described one pediatric case of ovarian torsion identified on POCUS through the pseudo-bladder sign, as well as four additional cases in which RADUS demonstrated the double-bladder sign. Although these signs indicate the presence of a pelvic cyst rather than torsion itself, they may serve as a diagnostic clue for ovarian torsion when accompanied by acute abdominal pain. Recognizing these findings, together with an understanding of normal pelvic anatomy and systematic scanning in both transverse and longitudinal planes, may facilitate earlier diagnosis and help avoid diagnostic delays or reduce the risk of ovarian loss. Further studies are needed to clarify their clinical significance and contribution to diagnostic accuracy.
